# *AIM* (Analyze–Interpret–Manage): A Novel NAPLEX-Aligned Analytical Assessment Framework for Measuring Individual and Team Critical Thinking Using Generative AI

**DOI:** 10.3390/pharmacy14010034

**Published:** 2026-02-12

**Authors:** Ashim Malhotra

**Affiliations:** Department of Pharmaceutical and Biomedical Sciences, California Northstate University College of Pharmacy, 9700 W Taron Drive, Elk Grove, CA 95758, USA; ashim.malhotra@cnsu.edu; Tel.: +1-(916)-686-8885

**Keywords:** critical thinking, NAPLEX competency, qualitative pharmacy education research, Delphi Report, AAC&U VALUE, cognitive team reasoning, discursive narratives, interprofessional education, IPEC

## Abstract

Critical thinking is emphasized across ACPE Standards 2025, the Pharmacist Patient Care Process, interprofessional education (IPE) frameworks, and licensure preparation (NAPLEX). Despite this, pharmacy education lacks a practical, theory-grounded framework that operationalizes critical thinking as an observable, assessable reasoning process, particularly in team-based and interprofessional contexts. We developed the AIM (*Analyze–Interpret–Manage*) framework by integrating the Delphi Consensus definition of critical thinking with the AAC&U VALUE framework, translating foundational theory into a concise, measurable, stage-based model applicable to both individual and collective cognition. AIM was tested using qualitative analysis of transcripts of student team discursive narratives of an assigned IPE scenario. Reasoning behaviors were coded by AIM stage and mapped to the 2016 IPEC Core Competencies and the 2025 NAPLEX competencies to ensure professional relevance and external validity. AIM reliably distinguished discrete stages of critical thinking across teams, revealing consistent patterns in how learners analyzed information, interpreted clinical and ethical significance, and managed decisions collaboratively. Mapping demonstrated strong alignment between AIM stages and IPEC and NAPLEX competencies. Our novel AIM framework offers a scalable approach for defining, teaching, and assessing team-based critical thinking in pharmacy education. By operationalizing critical thinking as a staged reasoning process aligned with professional standards, AIM fills a critical gap between educational theory, interprofessional practice, and licensure preparation.

## 1. Introduction

Critical thinking is universally recognized as a core outcome of pharmacy education and professional practice [1,2,3]. Accreditation standards, licensure expectations, and professional competency frameworks [4] consistently emphasize the ability of graduates to analyze information, integrate knowledge, and make sound, patient-centered decisions. The Accreditation Council for Pharmacy Education (ACPE) Standards 2025 explicitly identify critical thinking, clinical reasoning, and judgment as foundational expectations across curricular design, assessment, and outcomes evaluation [5]. Similarly, the North American Pharmacist Licensure Examination (NAPLEX) increasingly prioritizes application of knowledge, prioritization, and decision-making in practice-relevant contexts rather than factual recall alone [6].

Despite this centrality, critical thinking remains one of the least clearly defined and least consistently assessed constructs in pharmacy education [7]. While the term is frequently invoked in curricular goals, course objectives, and accreditation reports, there is limited consensus on what critical thinking *looks like in action*, how it should be observed, or how it can be evaluated in a manner that is both educationally rigorous and professionally relevant [8]. As a result, critical thinking is often inferred indirectly through examination performance, self-report instruments, or generalized reflective assignments rather than assessed as a visible reasoning process.

This challenge is not unique to pharmacy. Across the health professions, educational models such as the Flexnerian framework [9], characterized by foundational science instruction followed by clinical application, implicitly assume that learners will develop critical thinking through exposure and progression. However, evidence across disciplines suggests that learners frequently struggle to integrate foundational knowledge into clinical reasoning, particularly when required to interpret complex data, prioritize competing concerns, and translate analysis into actionable decisions [10,11,12]. The pharmacist patient care process (PPCP) [13] similarly presumes a progression from information gathering to assessment and care planning, yet it provides limited guidance on how reasoning within these stages should be examined or assessed.

Compounding this issue is the increasing emphasis on team-based and interprofessional care. Healthcare delivery is inherently collaborative, and many of the most consequential reasoning failures occur not at the level of individual knowledge deficits but at the level of communication breakdowns, role ambiguity, and collective decision-making [14,15]. The Interprofessional Education Collaborative (IPEC) Core Competencies (2016) [16] articulate expectations related to values and ethics, roles and responsibilities, interprofessional communication, and teamwork. However, like NAPLEX and PPCP, the IPEC competencies describe *outcomes* rather than the *reasoning processes* by which teams arrive at decisions.

The existing literature on critical thinking in pharmacy education reflects these conceptual ambiguities. Many studies focus on individual cognition, employ generic critical thinking instruments developed outside healthcare contexts, or rely on pre–post designs measuring knowledge gains rather than reasoning behaviors [17]. While valuable, these approaches often fail to capture how learners actually reason through authentic clinical and ethical problems, particularly in team-based settings. Moreover, they provide limited insight into how critical thinking aligns with licensure-relevant competencies or interprofessional practice expectations [18,19].

To address this gap, there is an urgent need for a framework that operationalizes critical thinking as an observable, staged reasoning process; aligns with professional competency structures such as NAPLEX and IPEC; and is applicable within authentic, team-based educational contexts. Such a framework should make explicit how learners move from information analysis to interpretation and ultimately to decision-making and action.

Here, we report the construction and feasibility testing of the novel AIM framework that operationalizes critical thinking as a set of three operatives: Analyze–Interpret–Manage.

**Figure 1:**
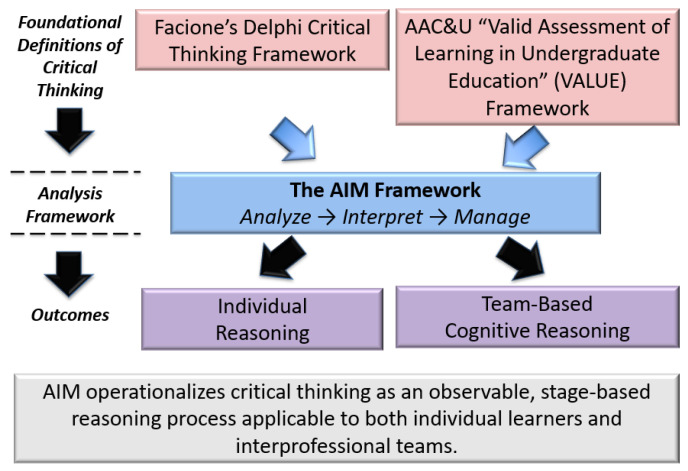
Conceptual foundations and development of the AIM (Analyze–Interpret–Manage) framework for critical thinking.

Our novel AIM (*Analyze–Interpret–Manage*) framework conceptualizes critical thinking not as a static trait or test score but as a dynamic process consisting of three sequential yet interrelated stages: (1) analyzing relevant information and standards, (2) interpreting meaning, implications, and priorities, and (3) managing decisions through justified action planning, as depicted in Figure 1. As detailed in Section 2, we utilized the two seminal foundational definitions of critical thinking to develop our analytical AIM Framework: (1) the Delphi Report on Critical Thinking [20] and (2) the AAC&U VALUE (Valid Assessment of Learning in Undergraduate Education) Framework [21]. Importantly, AIM is an operational and analytical framework that bridges the gap between the foundational definitions of what critical thinking “looks like” at the individual and team level and how the performance outcomes were reached by the individual or the team. AIM is designed to be applied at the level of observable discourse, allowing critical thinking to be examined through what learners say, prioritize, and propose during discursive narrative discussion, rather than inferred indirectly.

This study applies the AIM framework within an interprofessional education (IPE) setting, using student team discursive narrative discussions of a standardized clinical case as the analytic substrate. By mapping AIM-stage reasoning to both the IPEC Core Competencies [16] and the NAPLEX Content Outline [6], the study seeks to demonstrate how critical thinking can be assessed in a manner that is educationally rigorous, professionally grounded, and licensure-relevant. In doing so, this work aims to contribute a practical, theory-informed approach for defining, observing, and assessing critical thinking in pharmacy education.

## 2. Methods

### 2.1. Study Design and Conceptual Framework

This is an analytic feasibility study to explore conceptual coherence and illustrative application of the AIM Framework; it is not intended to be a psychometric validation. This study employed a qualitative, theory-informed analytic design to examine team-based critical thinking as demonstrated through interprofessional student discourse. The analytic approach was guided by the AIM framework (Analyze–Interpret–Manage), a novel, staged model of critical thinking developed for application in team-based healthcare education contexts.

The AIM framework was conceptually derived through the synthesis of two complementary traditions in the critical thinking literature: (1) Delphi-based definitions [20] of critical thinking, which emphasize cognitive processes such as analysis, interpretation, and inference, and (2) the AAC&U VALUE model [21], which emphasizes judgment, decision-making, and application in professional practice.

#### Theoretical Development of the AIM Framework

The AIM (Analyze–Interpret–Manage) framework was developed through an integrative synthesis of two complementary but traditionally separate approaches to critical thinking in the educational literature: (1) cognitive process-oriented definitions of critical thinking, and (2) performance-based assessment models designed to make complex reasoning observable.

First, the cognitive foundations of AIM were informed by the Delphi consensus definition of critical thinking, produced by an expert panel convened by the American Philosophical Association [20]. The Delphi framework conceptualizes critical thinking as a set of interrelated cognitive skills, including analysis, interpretation, inference, evaluation, and explanation, emphasizing disciplined reasoning at the level of the individual thinker [20]. While highly influential, Delphi-based models are primarily descriptive and do not specify how such reasoning may be observed, coded, or assessed in applied or team-based contexts [22].

Second, the operationalization of AIM was informed by the AAC&U VALUE Critical Thinking Rubric, which translates abstract reasoning constructs into observable indicators of performance [21]. The VALUE framework emphasizes developmental progression and assessment transparency, making it particularly suitable for educational evaluation. However, VALUE rubrics are not theory-generative and do not explicitly model the internal cognitive processes that underlie reasoning [23].

The AIM framework (Figure 1) was therefore intentionally constructed to bridge these two traditions. AIM’s *Analyze* and *Interpret* stages draw directly from Delphi-identified cognitive skills, while the *Manage* stage reflects applied decision-making and action planning in authentic professional contexts. By synthesizing cognitive theory with assessment-oriented operationalization, AIM provides a parsimonious framework capable of capturing both *individual reasoning* and *emergent team-based cognition*. This synthesis enables AIM to function as a theory-informed yet empirically usable framework for evaluating critical thinking within interprofessional educational settings.

The AIM framework was developed through the integration of two complementary traditions in the critical-thinking literature: (1) the Delphi Consensus model, which conceptualizes critical thinking as a set of core cognitive skills (e.g., analysis, interpretation, evaluation) at the level of the individual learner, and (2) the AAC&U VALUE framework, which operationalizes critical thinking through observable, assessable learning outcomes demonstrated in authentic academic work. AIM translates these foundational perspectives into a concise, stage-based reasoning model that captures three essential phases of critical thinking: (1) analyze, (2) interpret, and (3) manage. Importantly, the framework is designed to apply to both individual clinical reasoning and team-based cognitive processes, enabling its use in interprofessional education and collaborative healthcare contexts.

Thus, the AIM framework conceptualizes critical thinking as a progressive, observable process occurring across three stages: (1) *Analyze:* identifying, organizing, and prioritizing relevant information; (2) *Interpret:* assigning meaning to information through professional, ethical, and contextual lenses; and (3) *Manage:* proposing or justifying actions, decisions, or care strategies based on prior reasoning. This staged model was selected to align with how critical thinking is operationalized in healthcare practice and assessed in professional competency frameworks.

### 2.2. Alignment with Professional Competency Frameworks

**Figure 2:**
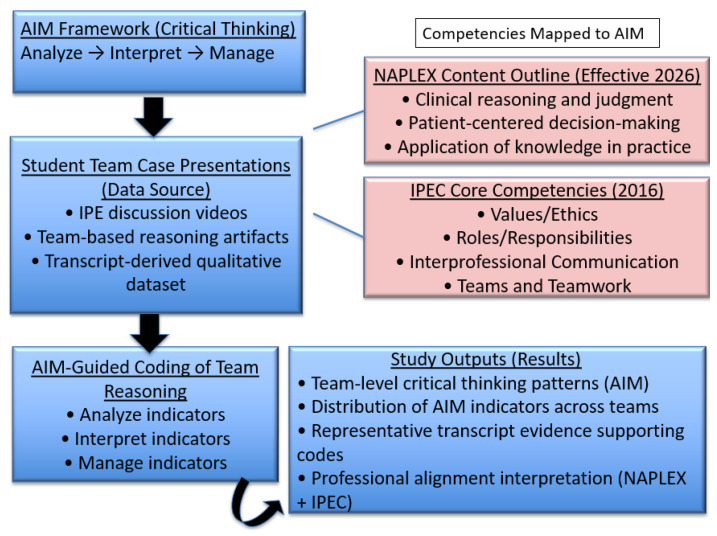
Application of the AIM framework to team-based critical-thinking analysis in an interprofessional education context.

To ensure professional relevance, regulatory alignment, and external validity, as explained in Figure 2, the AIM (Analyze–Interpret–Manage) framework was explicitly aligned with two nationally recognized competency structures used in pharmacy and interprofessional education: (1) the Interprofessional Education Collaborative (IPEC) Core Competencies for Interprofessional Collaborative Practice (2016), and (2) the North American Pharmacist Licensure Examination (NAPLEX) Content Outline (effective 2026). Although the author is aware that the IPEC Core Competencies have since been updated, the student assignment was conducted prior to the release of the updated competencies. Importantly, the more recent IPEC Core Competencies do not substantially differ from the 2016 version. IPE is used as a case example.

This figure illustrates the analytic workflow used in the present study. Team-based discussion transcripts were analyzed using the AIM framework to identify stage-specific reasoning behaviors (Analyze, Interpret, Manage). These reasoning processes were then mapped to two external competency frameworks: (1) the IPEC Core Competencies for Interprofessional Collaborative Practice (2016) [16] and (2) the NAPLEX Content Outline (effective 2026) to ensure professional relevance and external validity. Coding focused on patterns of reasoning, decision justification, and team cognition rather than factual correctness or clinical completeness. The resulting analytic outputs provide an operational approach for assessing critical thinking as an observable, team-based process aligned with professional competency expectations in pharmacy education.

Importantly, these frameworks were not treated as outcome checklists or used to evaluate factual correctness, clinical completeness, or therapeutic appropriateness. Instead, they served as interpretive anchors to examine *how* student teams demonstrated critical thinking through observable reasoning processes during case-based discussions.

#### 2.2.1. Mapping Logic and Unit of Alignment

Mapping was conducted at the level of cognitive and collaborative reasoning processes, rather than discrete content knowledge. Specifically, transcript segments were examined for evidence of: (1) problem identification and framing, (2) interpretation and prioritization of patient information, (3) justification of decisions using clinical, ethical, or professional reasoning, (4) coordination of roles and responsibilities across professions, and (5) collective decision-making and plan articulation. These reasoning behaviors were then aligned to AIM stages (Analyze, Interpret, Manage) and cross-referenced to relevant IPEC competency domains and NAPLEX blueprint categories.

This approach reflects how both IPEC and NAPLEX implicitly conceptualize competence: not merely as knowledge possession but as applied reasoning within professional contexts. For example, NAPLEX emphasizes application of knowledge, patient-centered decision-making, and clinical judgment, while IPEC emphasizes collaborative reasoning, communication, and ethical practice. AIM provides an intermediate analytical structure that makes these reasoning processes explicit and observable.

#### 2.2.2. Mapping Procedure

The alignment process occurred in three steps:

1. *Step 1—Framework Deconstruction*: The AIM framework was first operationalized into three distinct reasoning stages, each with observable indicators derived from transcript data. In parallel, relevant IPEC domains (2016) and NAPLEX blueprint elements (2026) were reviewed to identify overlapping expectations related to reasoning, decision-making, and professional judgment.
2. *Step 2—Cross-Framework Alignment:* Each AIM stage was mapped to corresponding IPEC competencies and NAPLEX domains based on conceptual congruence (e.g., interpretation of patient data aligning with clinical reasoning and interprofessional communication).
3. *Step 3—Evidence-Based Validation:* Mappings were verified through direct transcript evidence demonstrating that student teams engaged in reasoning behaviors consistent with all three frameworks simultaneously. This ensured that alignment was grounded in observed data rather than theoretical assertion.

A summary of this alignment is presented in Table 1, which illustrates how AIM functions as a unifying, practice-relevant framework bridging interprofessional education and licensure expectations.

### 2.3. Educational Context and Data Sources

Data were drawn from a required interprofessional learning activity involving pharmacy students working in small teams. The student-team activity was conducted at California Northstate University College of Pharmacy’s ACPE-accredited, four-year Doctor of Pharmacy (PharmD) program by 78 students in the P2 year. Students were divided into teams of 6–8 members. The activity was placed in the longitudinal practicum course, PRC709, and immediately preceded the start of interprofessional simulation and case conferences later in the semester. The objective of this activity was to familiarize PharmD students with the 2016 IPEC Core Competencies. Each team reviewed a standardized patient case (“Mrs. Robinson”) that was created with deliberately implanted errors. Student teams were tasked with producing a recorded, team-based discussion analyzing the quality of interprofessional care depicted in the scenario and were given one week to discuss and study the case, and another week to record their responses.

The recorded videos were uploaded to the instructor’s YouTube channel as unlisted videos for subsequent analysis. Student teams were instructed to reference relevant professional competencies (including the 2016 IPEC Core Competencies) during their discussions. Specifically, teams were instructed to examine and discuss whether the patient case contained sufficient information to analyze, interpret, and manage the case, especially with regard to the IPEC core competencies [16]. For example, teams were instructed to identify which IPEC core competencies remained unmet or were violated, explain the reason for their observation, and suggest one corrective measure, thus operationalizing the AIM framework.

The recordings were transcribed verbatim and de-identified prior to analysis. Each transcript served as a discrete analytic unit representing collective team cognition, rather than individual student performance. As noted, in addition to mapping student responses to the AIM framework, we also retrospectively analyzed their performance against the 2025 NAPLEX competencies [6] since these were mapped to AIM.

### 2.4. Qualitative Coding Strategy

A directed qualitative content analysis approach was employed. The AIM framework served as the a priori coding structure, with each transcript analyzed for evidence of reasoning aligned with the three AIM stages.

To preserve analytic clarity and feasibility, two primary indicators were defined for each AIM stage (six indicators total). Indicators were designed to be: (1) observable in spoken discourse, (2) applicable across teams, and (3) interpretable by non-education-specialist reviewers. Coding focused on what teams did cognitively (e.g., how they framed problems, justified interpretations, and proposed actions), not on whether their conclusions were clinically “correct.”

### 2.5. Analytic Procedure

Each transcript was reviewed iteratively. Segments of discourse were coded to AIM indicators when teams demonstrated: (1) explicit identification or prioritization of issues (Analyze), (2) interpretive reasoning linking observations to professional standards or ethical considerations (Interpret), or (3) action-oriented reasoning proposing, evaluating, or justifying care decisions (Manage). Coding emphasized patterns across teams, not frequency counts or individual utterances. Representative excerpts were selected to illustrate each indicator, with additional supporting evidence documented to demonstrate analytic saturation.

### 2.6. Large Language Model-Assisted Analytic Support

To support consistency and transparency in qualitative coding, analytic assistance was provided by a large language model (LLM), specifically ChatGPT (GPT-5.2, OpenAI, San Francisco, CA, USA). ChatGPT was used in a constrained, supportive role to assist with: (1) cross-team comparison of coded discourse, (2) identification of recurring reasoning patterns aligned with AIM indicators, and (3) verification of alignment between transcript excerpts and predefined analytic categories.

The model was not used to generate analytic categories, interpret findings independently, or replace investigator judgment. All coding decisions were theory-driven and finalized by the investigators. ChatGPT outputs were reviewed, curated, and selectively incorporated to enhance analytic consistency. All transcripts were de-identified educational artifacts, and no protected health information or interactions with human subjects occurred.

### 2.7. Rigor and Transparency

Methodological rigor was supported through: (1) explicit theoretical grounding of the AIM framework, (2) pre-specification of analytic indicators, (3) use of multiple teams to identify recurring reasoning structures, and (4) presentation of representative and supplementary transcript evidence to allow readers to evaluate interpretations independently. This approach prioritizes analytic transparency and reproducibility over statistical generalization, consistent with established qualitative research standards.

### 2.8. Reflexivity Statement

The author performed a dual role as both a framework developer and a framework analyst; his positionality was acknowledged a priori, and analytic decisions were guided by pre-specified indicators derived from theory. A second-pass audit by a faculty colleague was conducted to review coding logic and stage assignment. Inter-rater reliability was not a goal of this study, as the analytic aim was feasibility and conceptual coherence rather than reproducibility of scoring.

## 3. Results

### 3.1. Overview of Analytic Approach and Output

Student team transcripts (Teams 1, 4, 8, 12, 16, and 17) were analyzed using a directed qualitative approach guided by the AIM Framework, Analyze, Interpret, Manage, which operationalizes team-based critical thinking as a staged progression: (1) Analyze: identify and prioritize salient facts and standards in the case, (2) Interpret: explain significance, consequences, and meaning in context, (3) Manage: propose action steps, alternatives, urgency, or corrective decisions. Because the outcome of interest was demonstrated reasoning in discourse, the analysis did not rely on pre/post comparisons or descriptive statistics. Instead, analytic claims are supported by verbatim transcript evidence, and representative excerpts are presented to make the logic transparent and auditable for readers unfamiliar with qualitative coding. Across teams, evidence of all three AIM domains was observed, with *Analyze* and *Interpret* appearing consistently and *Manage* emerging more selectively and with greater variability in depth.

#### 3.1.1. AIM Domain 1: Analyze—Identification and Prioritization of Relevant Information

All teams demonstrated the ability to identify salient case elements (e.g., privacy breach, missed exam, poor follow-up timing) and associate them with professional standards, most often via explicit reference to IPEC competency language.

For example, Team 12 identified a privacy breach and named the ethical principle at issue: “The nurse failed to maintain the privacy of the patient by leaving medical information on the computer screen.” (Team 12).

Team 8 similarly isolated a clinical lapse (failure to assess abdominal pain) and labeled it as a competency failure: “The physician… does not check her lower abdomen… this is a violation of… competency number two…” (Team 8).

Across transcripts, *Analyze* was evident when teams: (1) isolated discrete breakdowns or successes in care, (2) categorized them as “met” vs. “not met,” and (3) mapped those observations to competency language or standards.

#### 3.1.2. AIM Domain 2: Interpret—Meaning-Making, Consequences, and Contextual Explanation

Multiple teams progressed beyond naming what happened to explain why it mattered, including patient safety implications, care quality consequences, and team-process breakdowns.

Team 1 interpreted the impact of a communication failure in terms of downstream patient care: “As a result her condition was not able to be treated.” (Team 1).

Team 4 interpreted the team-process implications of poor handoff communication: “If the nurse… communicate efficiently with the doctor… this whole process would have been… much more smoother.” (Team 4).

In these examples, teams moved from labeling competencies to contextual explanation. *Interpret* was demonstrated through: (1) causal explanations (because/therefore/as a result), (2) patient-centered consequence statements, and (3) interpretation of team function and breakdowns.

#### 3.1.3. AIM Domain 3: Manage—Actionable Judgment, Alternatives, and Urgency

Manage-level reasoning reflects the highest-order AIM domain: teams propose what should happen next (or what should have happened instead), including urgency, corrective actions, or better clinical/operational decisions.

Team 12 proposed a corrective action grounded in process and responsibility: “Mrs Robinson should have been examined by the physician… [through] communication between the nurse and the physician.” (Team 12).

Team 1 provided a clear urgency-based management judgment about follow-up timing: “With a blood pressure reading as high as 166 to 110, you shouldn’t be waiting a week… it should have been done immediately.” (Team 1).

*Manage*-level reasoning was evident when teams: (1) proposed alternative actions (“should have…”), (2) identified urgency/risk (“shouldn’t wait… immediately…”), and (3) recommended process corrections (handoffs, examinations, escalation).

Not all teams reached this level consistently, suggesting variability in the ability to translate interpretation into explicit action planning.

### 3.2. Cross-Team Pattern (Qualitative Summary)

Across all reviewed teams, Analyze and Interpret reasoning were observed consistently, while Manage reasoning appeared more unevenly. This pattern suggests that students readily identify and explain interprofessional issues, but fewer consistently articulate concrete next-step decisions unless prompted.

Taken together, these findings support AIM as a practical, reproducible lens for analyzing team-based critical thinking using narrative discourse in pharmacy education contexts.

To further substantiate these findings, Table 2 presents additional, non-duplicative transcript excerpts not previously cited in the narrative Results.

Table 3 provides further data from the recorded transcripts for analytic transparency for the application of the AIM framework, illustrating how student teams engaged in each stage of reasoning. Across multiple teams and discussion segments, statements coded as *Analyze* consistently reflected information gathering, fact clarification, and problem framing. *Interpret* excerpts demonstrated movement beyond description toward clinical meaning-making, ethical consideration, and prioritization of issues. *Manage* excerpts captured, collective decision-making, justification of action plans, and negotiation of next steps. Importantly, these excerpts were drawn from distinct moments across team discussions and are presented to demonstrate the observable and repeatable nature of the AIM stages rather than to evaluate correctness or performance. Together, the excerpts illustrate how the framework distinguishes qualitatively different forms of team reasoning and provides a transparent basis for mapping discursive behavior to professional competency expectations.

## 4. Discussion

### 4.1. Why a New Framework for Critical Thinking Is Needed in Pharmacy Education

Critical thinking is one of the most frequently cited [7] but least operationally defined outcomes in health professions education. Within pharmacy education specifically, accreditation standards (ACPE Standards 2025) [5], licensure expectations (NAPLEX) [6], and professional frameworks (PPCP [13], COEPA recommendations [4]) consistently emphasize critical thinking, clinical reasoning, and judgment. However, despite this rhetorical centrality, the field lacks a clear, observable, and practice-aligned framework for identifying how critical thinking is demonstrated, particularly in team-based and interprofessional contexts.

A review of the pharmacy education literature reveals that most published work addressing critical thinking relies on one of three approaches: (1) indirect self-report instruments [24], (2) standardized critical thinking tests developed outside healthcare contexts [20], or (3) pre/post designs assessing knowledge gains rather than reasoning processes [25].

While valuable for certain purposes, these approaches largely treat critical thinking as an individual cognitive trait or an outcome score, rather than as a process that unfolds through reasoning, interpretation, and decision-making in authentic practice settings.

The AIM (*Analyze–Interpret–Manage*) framework addresses this gap by reframing critical thinking as a staged, observable reasoning process, grounded in how healthcare professionals actually work. Rather than asking whether students “have” critical thinking, AIM asks whether students can: *Analyze* relevant information and professional standards, *Interpret* meaning, consequences, and priorities, and *Manage* decisions through action-oriented judgment.

Importantly, AIM does not replace existing competency frameworks. Instead, it functions as an analytic bridge that makes implicit reasoning expectations in IPEC and NAPLEX *explicit and assessable*. This distinction is critical: NAPLEX and IPEC define *what* competence looks like, but they do not specify *how* learners reason their way there. AIM fills that methodological and conceptual gap.

### 4.2. Why Interprofessional Education Is an Ideal Context to Examine Critical Thinking

A second contribution of this study is the intentional application of the AIM framework within an interprofessional education (IPE) context. Much of the critical thinking literature in pharmacy education focuses on individual cognition, often through written exams, individual reflections, or isolated simulations. However, contemporary healthcare practice is fundamentally collaborative, and many of the most consequential reasoning failures occur not at the level of knowledge deficits but at the level of communication, role clarity, and shared decision-making.

IPE provides a uniquely rich environment for examining critical thinking because it requires learners to: (1) integrate multiple professional perspectives, (2) negotiate meaning collectively, (3) justify interpretations to peers, and (4) translate reasoning into coordinated action [26].

These demands align closely with the higher-order stages of the AIM framework, particularly the *Interpret* and *Manage* domains. Our findings demonstrate that while students consistently identified issues (*Analyze*) and explained their significance (*Interpret*), fewer teams consistently articulated clear, actionable *management* decisions without prompting. This pattern mirrors known challenges in transitioning from knowledge integration to judgment and action, precisely the space where critical thinking is most consequential in real practice.

By situating AIM within IPE discourse rather than isolated individual performance, this study highlights an important conceptual shift: critical thinking in healthcare is not only an individual cognitive skill but a collective, dialogic process shaped by team dynamics and professional boundaries.

### 4.3. Implications for NAPLEX Readiness and Professional Competency Alignment

A notable strength of the AIM framework is its explicit alignment with licensure-relevant reasoning expectations. NAPLEX increasingly emphasizes application, prioritization, and patient-centered decision-making rather than factual recall. However, pharmacy programs often struggle to demonstrate how their assessments map onto these expectations beyond content coverage [27]. The AIM framework offers a practical solution by providing a reasoning-level analytic structure that aligns naturally with NAPLEX domains without collapsing into checklist-based evaluation. By mapping transcript-level reasoning behaviors to NAPLEX domains, this study demonstrates that licensure-relevant competencies can be examined through qualitative evidence of applied reasoning, not solely through exam performance.

This approach may be particularly valuable for programs seeking to: (1) demonstrate curricular alignment with NAPLEX beyond pass rates, (2) document higher-order reasoning development longitudinally, and support continuous quality improvement efforts required by accreditation.

### 4.4. Methodological Contribution: Demonstrating Qualitative Rigor Without Statistical Reduction

An anticipated concern among pharmacy education reviewers is the absence of traditional quantitative indicators such as pre/post scores or standard deviations. This study intentionally departs from that paradigm because the outcome of interest, *how teams reason*, cannot be meaningfully reduced to numeric change scores without losing explanatory power.

Instead, rigor is established through: (1) a theory-grounded analytic framework (AIM), (2) pre-specified indicators, (3) multiple independent team data sources, (4) transparent presentation of verbatim evidence, and (5) explicit alignment with external professional frameworks.

This approach aligns with best practices in qualitative health professions education research and responds directly to longstanding calls for methodological pluralism in pharmacy education scholarship. By making reasoning visible and auditable through transcript evidence, the study prioritizes interpretive transparency over statistical abstraction.

### 4.5. Future Validation and Applications of the AIM Framework

While this study establishes the feasibility and analytic utility of AIM, further work is needed to extend and validate the framework across contexts. Future studies could: (1) apply AIM longitudinally to track development across the curriculum, (2) examine AIM reasoning patterns in advanced experiential settings, (3) compare AIM-coded discourse with traditional performance metrics, or (4) explore faculty development applications for assessment calibration.

Importantly, AIM is designed to be discipline-agnostic within healthcare, making it adaptable to medicine, dental medicine, nursing, psychology, and allied health education. Its strength lies not in prescribing correct answers but in illuminating how learners *reason* toward decisions, a capability increasingly central to safe, equitable, and effective care. AIM is not intended to be used as a checklist in place of observing team critical thinking.

Although this study focused on pharmacy student teams, the analytic logic underlying AIM may apply to other healthcare contexts characterized by uncertainty, distributed expertise, and shared decision-making. Prior analyses of complex health system failures, such as medico-legal responses during the COVID-19 pandemic, have documented breakdowns in data interpretation, role clarity, and coordinated action, without providing a structured means to observe how such reasoning failures unfolded in practice. AIM offers a potential analytic lens for examining similar reasoning processes in future work, without presupposing discipline-specific content or outcomes [28].

#### Limitations

This study has several limitations. First, the analysis was conducted within a single institutional context using pharmacy students, which may limit transferability to other educational or professional settings. Second, qualitative coding was performed by the framework developer, introducing the possibility of interpretive bias despite the use of structured coding procedures. Third, the study was not designed to establish psychometric properties or inter-rater reliability, as its primary purpose was to demonstrate the analytic feasibility of the AIM framework. Finally, although transcript excerpts illustrate stage-specific reasoning, the qualitative design does not permit claims regarding prevalence, performance quality, or causal relationships among stages.

## 5. Conclusions

*Why Educators Should Use the AIM Framework.* Ultimately, the value of AIM lies in its practicality. It offers educators a shared language for discussing critical thinking, a structured lens for assessment, and a defensible method for demonstrating alignment with accreditation and licensure expectations. By operationalizing critical thinking as an observable process rather than an abstract ideal, AIM enables educators to move from aspiration to evidence.

## Figures and Tables

**Table 1:** Alignment of the AIM Framework with IPEC (2016) Core Competencies and NAPLEX (2025) Content Domains.

AIM Stage	Definition (Reasoning Focus)	Aligned IPEC Core Competencies (2016)	Aligned NAPLEX Content Domains (2026)	Illustrative Reasoning Behaviors Observed
***A—Analyze***	Systematic examination of patient information and contextual factors	Values/Ethics; Roles/Responsibilities	Domain 1: Patient-Centered Care; Domain 4: Population-Based Care	Identification of chief complaint; recognition of missing or unaddressed data; acknowledgment of privacy or safety concerns
***I—Interpret***	Synthesis and prioritization of information to define problems and implications	Interprofessional Communication; Values/Ethics	Domain 2: Safe and Effective Pharmacotherapy; Clinical Reasoning	Linking symptoms to clinical implications; identifying communication breakdowns; prioritizing patient safety issues
***M—Manage***	Development and justification of action-oriented decisions or plans	Teams and Teamwork; Roles/Responsibilities	Domain 3: Healthcare Systems; Decision-Making and Monitoring	Proposing follow-up actions; referrals; role-based interventions; collaborative care planning

Items in bold and italics represent the 3 stages of the proposed AIM framework.

**Table 2:** AIM Framework Evidence Table.

AIM Stage	Operational Definition	Observable Team Indicators (Codes)	Representative Transcript Evidence (Additional Evidence)
***A—Analyze***	Team identifies, parses, and prioritizes salient clinical and ethical information from a complex narrative.	Identification of key patient problems Differentiation of relevant vs. irrelevant details	Teams consistently isolated privacy breaches (e.g., unattended EMR screens) and unmet clinical assessments as primary issues before referencing competencies.
***I—Interpret***	Team meaningfully maps identified issues to professional standards, competencies, or frameworks.	3. Explicit linkage to IPEC competencies 4. Justification of why a competency was met or violated	Teams explicitly cited IPEC sub-competencies (e.g., VE2, RR5) and explained their applicability rather than listing them descriptively.
***M—Manage***	Team proposes or evaluates an action-oriented response consistent with scope of practice and team-based care.	5. Proposed corrective actions 6. Forward-looking care planning or referral decisions	Teams recommended improved interprofessional communication, immediate clinical reassessment, or appropriate referrals (e.g., pharmacist involvement) as corrective strategies.

Items in bold and italics represent the 3 stages of the proposed AIM framework.

**Table 3:** Analytic Transparency of AIM (Analyze–Interpret–Manage) Coding with Representative Verbatim Evidence.

AIM Stage	Coding Focus	How the Stage Was Operationalized	Representative Verbatim Transcript Evidence
Analyze	Identification of clinical problems	Explicit recognition of abnormal findings or omissions	*“The patient had very high blood pressure and H1AC; however, the physician only prescribed anti-inflammatory and pain medication.”*
Analyze	Recognition of missing assessments	Identification of absent physical examinations	*“The physician’s follow-up did not include a medical examination of her abdomen.”*
Analyze	Identification of safety risks	Recognition of urgency or potential harm	*“With a blood pressure reading as high as 166 to 110, you shouldn’t be waiting a week.”*
Analyze	Detection of process failures	Identification of communication breakdowns	*“There was no communication in a responsive and responsible manner.”*
Interpret	Assigning clinical significance	Explaining why identified issues matter	*“This shows a deficiency in competency, and her condition was not treated.”*
Interpret	Linking to standards	Connecting observations to competencies	*“This supports competency related to engaging other professionals.”*
Interpret	Prioritization	Ranking the severity of problems	*“There is a glaring deficiency… waiting a week is inappropriate.”*
Interpret	Role-based meaning	Interpreting professional responsibilities	*“The physician bringing in another healthcare professional was appropriate.”*
Manage	Action recommendation	Proposing corrective actions	*“She should have been treated immediately.”*
Manage	Care planning	Describing follow-up or management steps	*“I’ve already scheduled you an appointment.”*
Manage	Role-directed intervention	Assigning responsibility for next steps	*“The physician refers the patient to a clinical pharmacist.”*
Manage	Systems-level correction	Suggesting prevention of recurrence	*“This should have been done immediately rather than delayed.”*

## Data Availability

The original contributions presented in this study are included in the article. Further inquiries can be directed to the corresponding author.
